# The evolutionary dynamics of tRNA-gene copy number and codon-use in *E. coli*.

**DOI:** 10.1186/s12862-015-0441-y

**Published:** 2015-08-19

**Authors:** Michael J. McDonald, Chih-Hung Chou, Krishna BS Swamy, Hsien-Da Huang, Jun-Yi Leu

**Affiliations:** Institute of Molecular Biology, Academia Sinica, Taipei, Taiwan; Institute of Bioinformatics and Systems Biology, National Chiao Tung University, Hsinchu, Taiwan; Department of Biological Science and Technology, National Chiao Tung University, Hsinchu, Taiwan

## Abstract

**Background:**

The introduction of foreign DNA by Lateral Gene Transfer (LGT) can quickly and drastically alter genome composition. Problems can arise if the genes introduced by LGT use codons that are not suited to the host’s translational machinery. Here we investigate compensatory adaptation of *E. coli* in response to the introduction of large volumes of codons that are rarely used by the host genome.

**Results:**

We analyze genome sequences from the *E. coli/Shigella* complex, and find that certain tRNA genes are present in multiple copies in two pathogenic *Shigella* and O157:H7 subgroups of *E. coli*. Furthermore, we show that the codons that correspond to these multi-copy number tRNA genes are enriched in the high copy number Selfish Genetic Elements (SGE’s) in *Shigella* and laterally introduced genes in O157:H7. We analyze the duplicate copies and find evidence for the selective retention of tRNA genes introduced by LGT in response to the changed codon content of the genome.

**Conclusion:**

These data support a model where the relatively rapid influx of LGT genes and SGE’s introduces a large number of genes maladapted to the host’s translational machinery. Under these conditions, it becomes advantageous for the host to retain tRNA genes that are required for the incorporation of amino acids at these codons. Subsequently, the increased number of copies of these specific tRNA genes adjusts the cellular tRNA pool to the demands set by global shifts in codon usage.

**Electronic supplementary material:**

The online version of this article (doi:10.1186/s12862-015-0441-y) contains supplementary material, which is available to authorized users.

## Background

Laterally Transferred Genes play an important role in the adaptation of bacterial pathogens [1–5]. Most genetic changes arising from LGT, especially insertion of SGEs, will be deleterious or neutral [6, 7]. However, laterally transferred pathogenicity islands in *E. coli* and other pathogens show that large groups of laterally transferred genes can fix in bacterial populations [8–11]. Similarly, SGEs can proliferate to the extent that they comprise a significant proportion of the genomes of some bacteria. SGE’s and LGT genes can contain codons that are rarely found in the host genome. As such, it is of interest to investigate the indirect effects of introducing large numbers of these genes upon genome level traits such as codon usage and the global tRNA pool.

The elevated expression of a gene with low CAI (Codon Adaptation Index) codons can have a strong impact on growth rate [12–15]. Translation is the most energetically demanding of cellular processes, and the presence of many genes with poorly adapted codons could lead to strong selection for better adapting the genes to translation in the host. Indeed, LGT seems to occur most readily between genomes that use similar codons [16, 17]. The favored model by which a gene becomes better adapted to its host genome is by the stepwise fixation of substitutions that change rarely used codons into preferred codons [18]. This is supported by between species comparisons that find that the degree of codon adaptation increases for individual genes or operons that have higher expression [19].

However, there are reasons why this process maybe less effective for larger volumes of DNA. First, because the number of sites that requires replacement could be very large, the step-wise replacement of individual sites would take a prohibitively long time. Second, the selective advantage conferred by the conversion of a single codon site will be very small and thus the path to fixation would be sinuous and prolonged.

An alternative solution is that, instead of changing the codon content of many genes, the tRNA gene content of the cell can change [16]. There are good reasons why this might be plausible. The duplication of a tRNA gene is a relatively common mutational event [20], and would simultaneously effect all genes containing the non-optimal codons corresponding to that tRNA gene. Moreover, increasing the copy number of a tRNA gene has been demonstrated to increase the amount of the tRNA in the cell [21, 22], a correlation partially due to tRNA being a direct product of transcription. In support of this hypothesis, Tuller and Co-workers found that genes that were more recently introduced into the genome via LGT contained codons that were less well suited to the genomes set of tRNA genes[16], indicating the presence of selective pressure to change the tRNA content of the cell. This was further supported by the calculation of an “optimal” set of tRNA genes for a given set of genes: the authors found that the genome wide set of genes had a better fit that the set of recently introduced genes [16].

The vast repository of bacterial genome sequences provides a superb resource for addressing questions of genome evolution. A popular approach is to search for trends by analyzing a representative sample of all sequenced bacterial species. However, these sets of species can span a greater evolutionary distance than all of *Eukarya*, and such large divergence times can obscure patterns of interest. As an alternative, we look at a model set of strains within the *E. coli*/*Shigella* complex [23], ideal because of the high quality of its sequenced genomes and the extensive knowledge of their genetics [23–25]. The range of divergence for these strains is 0.1–2.4 %, allowing us a “shutter speed” sufficient to capture events that can elude studies focusing on between-species comparisons. Here we exploit this model system to investigate the genome level response to influxes of foreign DNA.

*Shigella* strains have been known to acquire large numbers of SGEs [26], and the pathogenic O157:H7 strains of *E. coli*, have sustained the introduction of many genes by LGT [10]. We find evidence that suggests that pathogenic strains of *E. coli* have adapted to changes in codon use by increasing the copy number of key tRNA genes. Our results suggest that this is achieved by the selective retention of tRNA genes introduced by phage mediated LGT.

## Results and Discussion

### tRNA genes are over-represented in *Shigella* and O157:H7 genomes, but not other *E. coli* strains

**Fig. 1:**
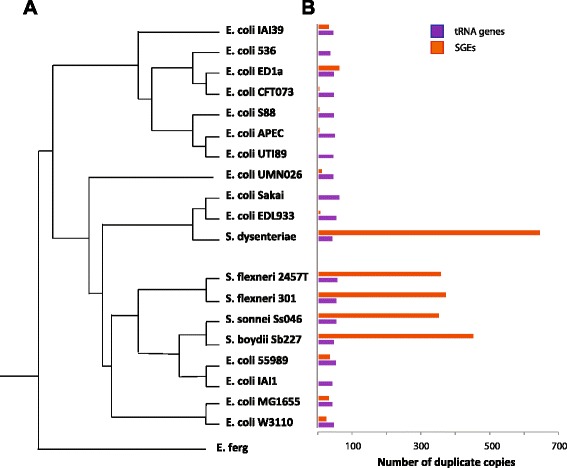
tRNA and SGE genes frequently have multiple copies in *E. coli*. **a**. The phylogenetic relationship of the *E. coli* strains used in this study, as established by Touchon et al. 2009. **b**. The bars show the numbers of duplicate copies of tRNA genes and SGE genes in each *E. coli* strain

**Fig. 2:**
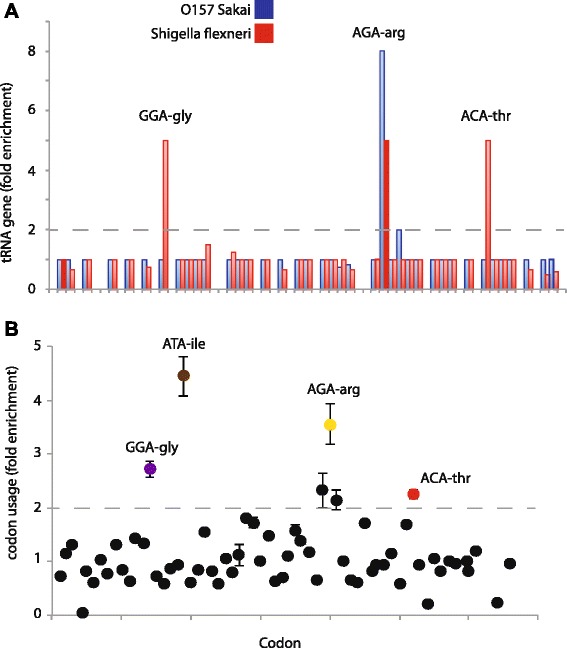
The codons enriched in Shigella SGE’s correspond to high copy number tRNA genes. **a**. The numbers of each tRNA gene, relative to *E. coli* MG1655. One representative strain of the O157:H7 strains (*Sakai, blue*) and *Shigella* strains (*S. flexneri* 2A 2457T, *red*) are shown for clarity, see Additional file 2: Figure S1 for the results using other *Shigella* and O157:H7 strains. Significant (greater than two fold enrichment, Mann-Whitney’s U) tRNA genes are labeled. **b**. The relative use of codons in the set of SGE elements that have multiple copies in *Shigella* strains. Each point shows the average enrichment of that given codon among the five *Shigella* strains used in this study, error bars are 99 % confidence intervals. Significance was calculated using Mann-Whitney’s U (colored circles)

We scanned *E. coli* genomes (Table 1) for the copy number of all tRNA genes for each of the 64 anticodons (Fig. 1). Many tRNA genes showed copy number variation, typically plus or minus one copy compared to other strains (Additional file 1: Table S1). Two subgroups, O157:H7 (Sakai and EDL933) and *S. flexneri* (Sf301, Sf2457T, Sb227, Sd197 and Ss046) had at least a 2-fold enrichment for select tRNA genes, compared to the non-*Shigella* or O157:H7 *E. coli* strains. For O157:H7 these were tRNA genes corresponding to AGA-arg, and for *Shigella*, GGA-gly, ACA-thr and AGA-arg (Mann–Whitney U, *p* < 0.005) (Fig. 2). The most striking example from the above analysis is the multiple copies of a tRNA^AGA^ gene, which in the type strain MG1655 is encoded by a single copy of *argU*, while O157:H7 Sakai has eight tRNA^AGA^ genes.

**Table 1:** Strains used in this study

Ref seq ID	Strains
NC_000913	Escherichia coli str. K-12 substr. MG1655
AC_000091	Escherichia coli W3110
NC_002655	Escherichia coli O157:H7 EDL933
NC_011353.1	Escherichia coli O157:H7 EC4115
NC_013008.1	Escherichia coli O157:H7 TW14359
NC_002695	Escherichia coli O157:H7 Sakai
NC_013941.1	Escherichia coli O55:H7 CB9615
CP003109.1	Escherichia coli O55:H7 str. RM12579
NC_007613	Shigella boydii Sb227
NC_007384	Shigella sonnei Ss046
NC_004337	Shigella flexneri 2a str. 301
NC_004741	Shigella flexneri 2a str. 2457T
NC_008563	Escherichia coli APEC O1
NC_007946	Escherichia coli UTI89
NC_004431	Escherichia coli CFT073
NC_008253.1	Escherichia coli str. 536
NC_011740	Escherichia fergusonii ATCC 35469
NC_011741	Escherichia coli IAI1
NC_011742	Escherichia coli S88
NC_011745	Escherichia coli ED1a
NC_011748	Escherichia coli 55989
NC_011750	Escherichia coli IAI39
NC_011751	Escherichia coli UMN026
NC_007606.1	Shigella dysenteriae sd 197

Over expression of *argU* has been shown to facilitate expression of genes containing its rare anticodon AGA [12, 13], and a deficiency of *argU* can lead to frame shifts in such genes [27]. These experimental studies suggest that selective pressure on genomes to maintain sufficient levels of tRNA anticodons could be substantial. We next sought to see if this change in the tRNA gene complement of the cell was reflected in the DNA content changes detected in these strains in the gene copy number analysis.

## Systematic analysis of repeat elements across *E. coli* reveals the enrichment of SGEs in *Shigella*

We found that the genes the comprise the Insertion Sequences *IS1*, *IS2* and *IS600* were the most prolific SGEs in *E. coli* genomes (Fig. 1b). Selfish elements are more numerous in the *Shigella* strains, compared to non-*Shigella E. coli* (*t*-test, *p* = 0.0015), with each *Shigella* strain having on average 420 more transposase, integrase or transposon repressor genes than non-*Shigella E. coli* strains (Table 2).

**Table 2:** The average number of selfish genetic elements in *Shigella* strains

Function	Average *shigella*	Average
		*E. coli*
IS 600 integrase	45.4	1.1
IS600 transposase	45.4	0.7
IS2 repressor tnpA	32.6	1.5
IS2 transposase	32.4	1.6
IS1 repressor	141.0	3.0
IS1 transposase	141.0	3.0

The SGE component of *Shigella* genomes, about 440 genes, makes up a significant proportion of the genome. Despite the swelling of the genome due to SGE proliferation, the genome sizes of *Shigella* (4.3–4.6Mb) has not increased relative to phylogetically close *E. coli* strains, thus SGE comprise approximately one tenth of the genome by gene number. Because these *Shigella* strains are phylogenetically dispersed across *E. coli*, it is unlikely that the apparent enrichment of SGE elements in *Shigella* strains is due to non-*Shigella* strains having experienced a systematic decrease in SGE insertions. The fixation of IS elements in *Shigella* could be beneficial, but is quite possibly deleterious or neutral and occurs due to genetic drift [28]. Whether a new IS element insertion is beneficial or deleterious, once it is fixed in the population and expressed, the cell will be under selective pressure to mitigate any deleterious effects of it’s insertion, or the insertion of multiple IS elements.

## *Shigella* selfish elements display differential codon usage that corresponds to the enriched tRNA genes

We analyzed the codon usage of the *Shigella* SGE genes, and then calculated the fold enrichment of each codon compared to the genome wide average, and did this for each *Shigella* genome (Fig. 2). Using a cut-off of 2-fold enrichment we found that codons corresponding to AGA-arg, ACA-thr and GGA-gly, the most enriched tRNA genes, were also enriched in the selfish elements compared to a matched set of genes randomly chosen from the *E. coli* genome (Mann–Whitney U, *p* < 0.0001). However, we found another codon (ATA-ile) that is frequently used in the coding sequence of *Shigella* SGEs (4-fold enrichment, Mann–Whitney U, *p* < 0.0001) while the tRNA^ATA^ gene was not found in multiple copies in any genome. This could be that the tRNA^ATA^ gene had increased its expression by means other than gene duplication, or even that genes containing high levels of ATA codons do not suffer a detrimental effect on expression. Even allowing for the ATA-ile codon, that three of the four most enriched codons corresponded to the three tRNA genes with the highest copy number is significant (Permutation test, *p* = 0.003, see Methods).

We counted the absolute numbers of codons, finding that on average 17,089 AGA-arg, 26,292 GGA and 17,966 ACA-thr codons are introduced into *Shigella* genomes by SGE proliferation. These results, together with previous experiments demonstrating the cost of expressing genes requiring rare tRNAs suggest that it is plausible that the proliferation of tRNA^AGA^, tRNA^GGA^ and tRNA^AGA^ genes maybe a selective response to the stress applied by the massive shift in codon usage demanded by the gain of 440 genes, using codons rarely used by the host genome.

## LGT genes are enriched with AGA codons in proportion to the copy number of the tRNA^AGA^ gene “*argO*” in O157:H7

**Fig. 3:**
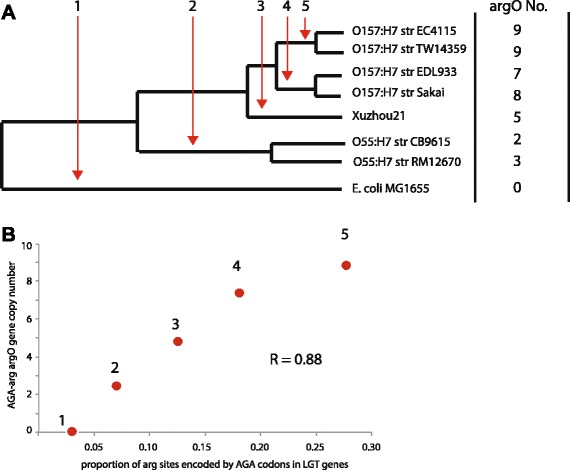
The enrichment of O157:H7 strains correlates with the tRNA^AGA^ gene copy number. **a**. A phylogeny of sequenced O157:H7 strains (Methods). Numbers above the red arrow indicate where putative LGT genes are binned as occurring before the divergence of the node indicated. The column to the right shows the number of tRNA^AGA^ genes that can be found in each genome. **b**. The number of tRNA^AGA^ genes is plotted against the proportion of all arginine sites encoded by AGA in the LGT originating genes. The exact numbers used for plotting this figure can be found in Table 3. The genes corresponding to nodes 2, 3, 4 and 5 are provided in supplementary data

If the increase in tRNA gene number is a selective response to increased codon use, then the numbers of tRNA genes and codon use should be correlated. The O157:H7 lineage is known to have sustained many LGT events during its history [10], and does not have an unusual enrichment of selfish elements, so we started by examining the LGT content and codon use of an expanded set of O157:H7 strains (Table 1, Fig. 3a). We used a BLAST-based phylogenetic method (see Methods) to create a list of LGT genes that are specific to the O157:H7 clade and not other *E. coli* strains. We then determined which of the total set of O157:H7 LGT genes were specific to each clade within the O157:H7 phylogeny at the point at which that clade diverged from the common ancestor shared with Sakai (see Methods). We compared the codon use of these subsets of laterally transferred genes to the number of *argO* copies (see Methods). For all O157:H7 strains, the most duplicated tRNA gene corresponded to the most enriched codon (AGA-arg) in laterally transferred DNA. We found that there was a strong correlation between codon usage and tRNA gene copy number in all O157:H7 strains (*R* = 0.88, *p* = 0.0042)(Fig. 3b, Table 3).

**Table 3:** Codon usage and tRNA^AGA^ copy number for subsets of laterally transferred genes in O157:H7 strains.

LGT event	AGA codon use	tRNA no
0	0.030	0
1	0.069	2.5
2	0.125	5
3	0.180	7.5
4	0.276	9

## The additional copies of tRNA genes are introduced by LGT and not by within-genome duplication

There are two plausible models of tRNA gene copy number increase. The first is that genes are duplicated within the genome during DNA replication or recombination mediated repair. This will result in two identical copies of the gene, and usually a surrounding syntenic region of DNA. Alternatively, additional copies of genes can be reintroduced by LGT. In this case additional copies are less likely to be identical, and maybe flanked by phage-associated genes, since phage mediates many LGT events. We thus sought to compare these two models of gene copy acquisition.

LGT is highly frequent in bacterial genomes [1, 5]. If the probability that one individual will contribute DNA via LGT to another is influenced by proximity, it seems likely that most LGT events must originate in the same species or genus. Moreover, LGT is hypothesized to often occur during phage infection [29]. Since phage has restrictions on the range of hosts that they can infect, it seems likely that new genes that are introduced via LGT could be identical to genes already present in the host. In such cases it would be very difficult, or perhaps impossible to distinguish between LGT events and within-genome duplication events by a post hoc analysis. Nonetheless, in some cases it should be possible to distinguish between these two hypotheses.

**Fig. 4:**
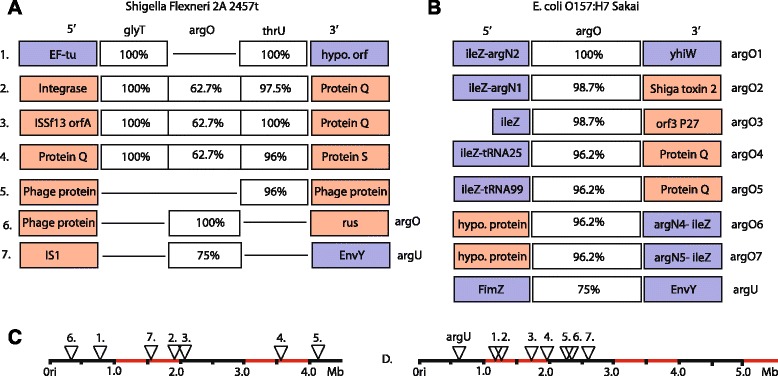
Multicopy tRNA^AGA^ genes of *Shigella* and O157:H7 and their genomic context. **a**. The *glyT* and *thrU* genes of *S. flexneri* 2A 2547T often occur together, however the DNA sequence immediately flanking the insertion site of additional copies are flanked by phage (*orange boxes*), rather than bacterial genes (*purple boxes*). Closest flanking genes were determined by genome specific BLAST search. Percentages indicate degree of sequence identity to the gene designated as the original host copy **b**. The tRNA^AGA^ genes *argU* and *argO* of O157:H7 Sakai. Percentages indicate the sequence identity to the gene annotated as argO1 in the O157:H7 Sakai genome. **c** and **d**, The positions of tRNA gene insertions across the genome for *Shigella flexneri* 2A 2475 t and O157:H7 Sakai. Precise numbers and sites of insertion vary for other members of the O157:H7 and *Shigella* subgroups

The mechanisms of within-genome gene duplication often result in the formation of tandem arrays of identical duplicate copies. This can be observed for some sets of tRNA genes that are present in high copy numbers. For example the *glyV*, *glyW*, *glyX* and *glyY* tRNA genes are present in a tandem array of three or four identical copies in all *E. coli*. We found that a tandem arrangement was not evident for any of the tRNA genes that we have described as enriched in *Shigella* or O157:H7 strains (Fig. 4). Interestingly, in *Shigella* the *glyT*, *argO* and *thrU* genes, all of which were present in high copy numbers in *Shigella*, were found together in multiple instances. Each of these copies, except for the original, were flanked by phage sequences suggesting that these genes were introduced by phage mediated LGT. It is plausible that there was a single phage mediated integration event and then this was duplicated by within-genome duplication events. However, recently duplicated genes should show a high degree of sequence identity and while the sequence of *glyT* showed perfect sequence identity across all copies, *argO* and *thrU* showed a relatively large degree of sequence variation, suggesting that duplicate copies had time to diverge (Fig. 4).

The O157:H7 cluster of strains contain between 4 and 9 copies of tRNA^AGA^ genes. Each O157:H7 strain has a single copy of *argU*, as do all *E. coli* genomes, with all additional tRNA^AGA^ genes being *argO*, a phage originating tRNA gene with only 75 % sequence identity with *argU* (Fig. 4b). It is possible that, although this gene is phage in origin, after its initial introduction via LGT that gene duplication contributed to the proliferation of this gene. However, the lack of tandem arrays of this gene and the association of each insert with flanking phage genes suggest that each copy originated from outside the genome. Two pairs of *argO* genes, *argO4*/*argO5* and *argO6*/*argO7* appear to be the result of duplication events due to the synteny of the surrounding genes. However, *argO6* and *argO7* have only 96.2 % sequence identity. The only two identical copies of *argO, argO4* and *argO5,* are the most promising candidates for within-genome duplication. While it is possible that one or two of the seven *argO* copies are due to a within-genome duplication event, it seems that the retention of genes introduced by LGT plays a more important role in increasing gene copy number for tRNA genes both in *Shigella* and O157:H7.

## Conclusion

**Fig. 5:**
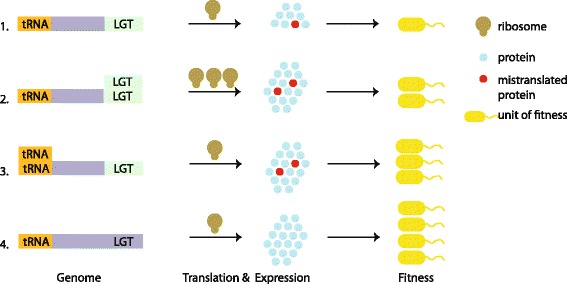
A step-wise model of compensatory adaptation to LGT. 1, LGT DNA (*green box*) inserts into the genome and causes an increase in fitness, fixing in the population. 2, Expression of the gene is hampered by non-optimal codon use. In order to increase the expression of the LGT gene, LGT DNA is duplicated (green boxes) [38, 39], causing an increase in fitness. This comes at the cost of the inefficient use of the translational machinery, here shown by increased use of ribosomes. 3, tRNA genes (*orange box*) are duplicated, resulting in more efficient translation of the LGT genes, and reduced demand upon the translational machinery. 4, The LGT genes become integrated, adjusting codon use by way of mutational pressure and selection for lower translation error and missense mutation rates [35]. Purple indicates integrated host DNA

The picture of tRNA gene copy number and codon use evolution has become more sophisticated over decades of study. Early workers concluded that codon usage is constrained by the tRNA genes present in the genome [30, 31], while others have posited theoretical models concluding that tRNA copy number and codon usage could coevolve [32–35]. The work presented here tentatively supports that the set of tRNA genes can change in response to drastic changes in codon use. Is the change in tRNA number a long-term evolutionary solution to changes in genome composition of *Shigella* and O157:H7? Other work has shown that tRNA usage is under constraints enforced by GC mutational biases and missense errors inherent to certain tRNA-codon pairs [35]. If this is indeed the case, then the increase in tRNA gene copy number observed here may only be a single step in a cascade of possible evolutionary steps initiated by LGT (Fig. 5).

## Methods

### Dataset collection

We obtained 23 genomes of strains of the *Escherichia coli/Shigella* complex strain from NCBI Genome databases. Accession numbers and names shown in Table 1.

### Gene copy number analysis

*E. coli* MG1655 gene annotations were used as a reference for identifying genes with multiple copies within its own genome and 19 other *Escherichia coli/Shigella* strains (Table 1). First, MG1655 genes were blasted against its own genome, with an identity cutoff of >95 % and gene length coverage >95 %. This cutoff was selected as it exceeds the maximum average divergence between any two strains that we analyzed, thus ensuring that we detect all duplications happening during divergence of the *E. coli/Shigella* complex. A consequence of this is that we found that many of the duplicated genes have paralogs that had arisen by duplication more recently that 5 % divergence, yet were present in multiple strains. In order to avoid mistaken assignation of GO identity these paralogous genes were clustered into groups. Using *E. coli* MG1655 *asnT* gene as a blast query gene for example, we can get hits for *asnT* as well as *asnU*, *asnV* and *asnW*. And when we choose *asnU* as a blast query gene, the results also shows *asnU* can align with *asnT*, *ansV*, and *asnW*. After using blast to define the entire gene cluster, we use MG1655 gene cluster group to find duplicated gene in others strains genome. For example, MG1655 *asnT*, *asnU*, *asnV*, and *asnW* are a gene cluster. If another strain contains any one of these four genes, we use their own gene to blast search duplication. In the case of Insertion Sequence genes, copies that had slightly difference sequence identities could result in erroneous independent blast hits, thus inflating the apparent number of SGE “sets”. In order to overcome this, the larger SGE sets were verified by manually blasting an individual within a set of duplicates and looking at the position and number of its sister duplicates. If this matched another set of duplicate genes that had been uncovered in the analysis one of these sets was culled from the analysis, resulting in a high-confidence, conservative set of duplicate gene sets.

### tRNA gene and copy number analysis

Small sequence differences between tRNA genes can specify different codon specificity, and tRNA genes that exhibit great variance can specify the same amino acid. In order to accurately determine the codon specificity for each tRNA gene, genbank files were extracted to obtain known codons and a tRNA search tool “tRNAscan-SE 1.3” [36] used to find and identify the specificity of all tRNA genes. The “fold” increase of a given tRNA gene in a given genome was calculated by dividing the number of that tRNA gene in the genome divided by the number of that tRNA gene in the ancestral genome, taken to be *E. coli* K12 MG1655. The significance of fold increase was calculated by first dividing all strains used in this study into three sets: *Shigella* (Sf301, Sf2457T, Sb227, Sd197 and Ss046), O157:H7 (Sakai, EDL933, EC4115, TW14359, Xuzhou21, CB9615 and RM12579) and all others (MG1655, W3110, APEC 01, UT189, CFT073, 536, ATCC 35469, IAI1, ED1a, 55989, IAI39, UMN026). The mean fold change for a given tRNA gene was calculated for individual genomes, and fold changes greater than two fold were tested for significance by using the non-parametric Mann–Whitney *U* test to compare either the “*Shigella*” or “O157:H7” set with the “others” set.

### Codon usage analysis

In order to determine codon usage throughout each genome, we developed a program that scans the in-frame triplets for each open reading frame, assigning an amino acid to each triplet as specified by the genetic code. All of the codons that code the same amino acid will add up to 1, with each codon taking up a proportion based on its absolute frequency of occurrence in the genome. For example, the amino acid alanine has in *E. coli* four codons which code for its incorporation into polypeptides: GCG, GCC, GCA, GCT. Each of these codons was assigned frequencies of 0.36, 0.27, 0.21 and 0.16 respectively. Because, the bacteria start codon can be encoded by a variety of triplets, here all of the start codons are considered as methionine when calculating codon usage.

### Calculating the degree of enrichment of codons in laterally transferred genes compared to all genes

In order to compare codon use in laterally transferred genes to all other genes, we created a list of all the genes in the focal genome as well as all of the laterally transferred genes in the focal genome. Individual codons were compared by calculating the proportion of increase or decrease experienced by that codon in the laterally transferred genes compared to all genes. Because the set all genes included laterally transferred genes, this is a conservative measure of the change in codon use found in the sets of laterally transferred genes. For example, for the AGA-arg codon in Sakai, the codon use in the set of all genes was 0.03, meaning that for the entire genome, only 3 % of Arginine sites in proteins were encoded by AGA. However, the codon usage in the laterally transferred genes was 0.23 meaning that 23 % of arginine sites in proteins were encoded by AGA in genes of foreign origin. In order to obtain a measure of the change in laterally transferred genes compared to all genes we divide 0.23 by 0.03, giving an enrichment of 7.9 fold in LGT compared to all genes. We performed this calculation for each codon, and then ranked codons from the most to least enriched in laterally transferred genes. In order to test the hypothesis that the codons corresponding to duplicated tRNA genes were more highly ranked than expected we compared the observed ranking to that expected under the null hypothesis of random rankings. This was done by summing the ranks of the codons corresponding to the three duplicated tRNA genes, for example as seen in Fig. 2b, the AGA, GGA and ACA codons were ranked 2nd, 3rd and 4th most enriched, giving a combined total of “9”. It is expected that if tRNA genes are duplicated because of the increased usage of their corresponding codon this sum of ranks should be lower than expected by chance. We generated 10,000 random sets of three numbers between 1 and 64 and used this as the null expectation for how often we could expect a sum of ranks less or equal to the observed value. P values were calculated based on how many of the randomly generated sets of three numbers had a sum less than or equal to 9. Because this test was designed specifically to examine one tail (the higher ranks) of the distribution of events, this test is 1-tailed.

### LGT gene analysis

We used a phylogenetic tree built from 30 sequenced *E. coli* genomes, including all the O157:H7 strains used in this study. This tree was consistent with another tree built using 341 non-recombingenic genes [37]. Confident that this provided a robust O157:H7 phylogeny, we sought to first identify LGT genes unique to this clade within *E. coli*. We used the O157:H7 str. Sakai strain as a starting point and searched all bacteria and phage for the best possible hits to each of its genes (excluding the closely related O157:H7 strains EDL933, EC4115, TW14359, Xuzhou21, CB9615 and RM12579). This allowed us to identify a conservative set of laterally transferred genes there were present in O157:H7 str. Sakai and closely related strains listed above, but not in the rest of *E. coli*. The presence of each of these genes could be due to LGT events that could have occurred at any point after the divergence of the common ancestor of all the O157:H7 strains from the rest of *E. coli*. This resulted in a set of 322 genes whose closest ancestor outside of O157:H7 was found outside of *E. coli*. We next looked for the presence or absence of each of these 322 genes in each of the seven O157:H7 genomes used for this analysis. The allowed us to assign subsets of the 322 genes to subclades within the tree. The codon usage of these subsets of genes were compared to the number of AGA-arg tRNA genes present in the branch tip species using phylogenetic independent contrast to correct for the nonindependence of data points resulting from phylogeny (R package, “ape”).

## Availability of supporting data

The data sets supporting the results of this article are included within the article (and its additional files), accession numbers for all genomes used in this study are available in Table 1.

## Additional files

Additional file 1: Table S1. The tRNA gene copy number for major* E. coli *strains used in this study. (XLSX 41 kb) Additional file 2: Figure S1. Enriched tRNA genes for O157:H7 Edl933 concur with the tRNA genes enriched in O157:H7 Sakai (Fig. 2a). Enriched tRNA genes for all other *Shigella* strains concur with the tRNA genes enriched in O157:H7 Sakai (Fig. 2a). (PDF 847 kb)

## Abbreviations

SGE: Selfish genetic element
LGT: Lateral genetic transfer
GO: Gene ontology
