# Supplementary figures and images for: The evolutionary dynamics of tRNA-gene copy number and codon-use in E. coli
Source: BMC Evol Biol. 2015 Aug 19;15:163. doi: 10.1186/s12862-015-0441-y (PMC4539685; doi:10.1186/s12862-015-0441-y)

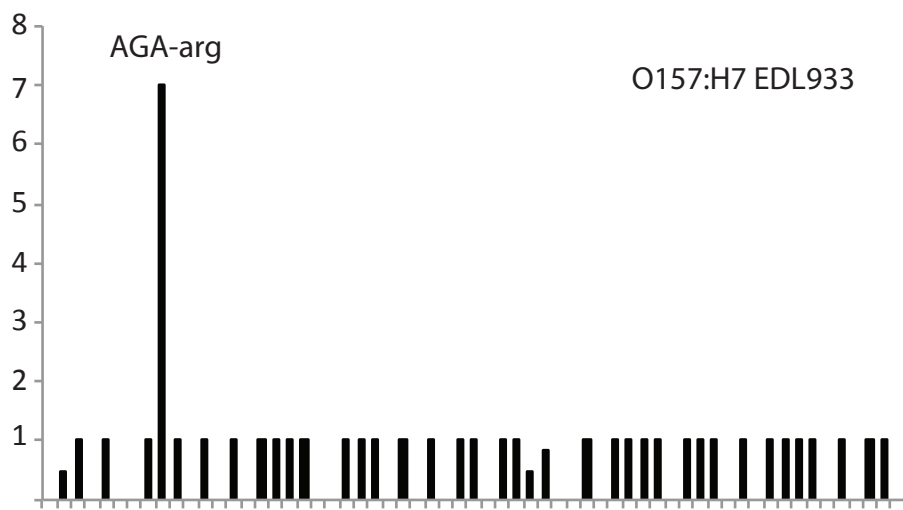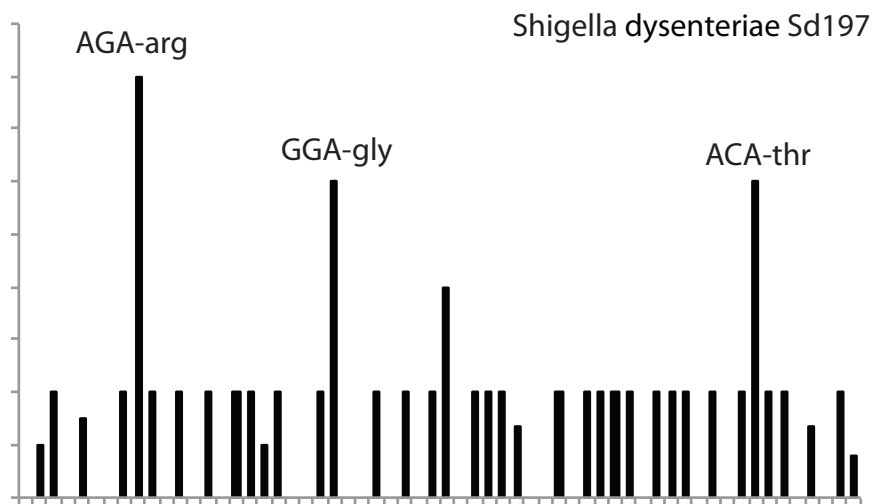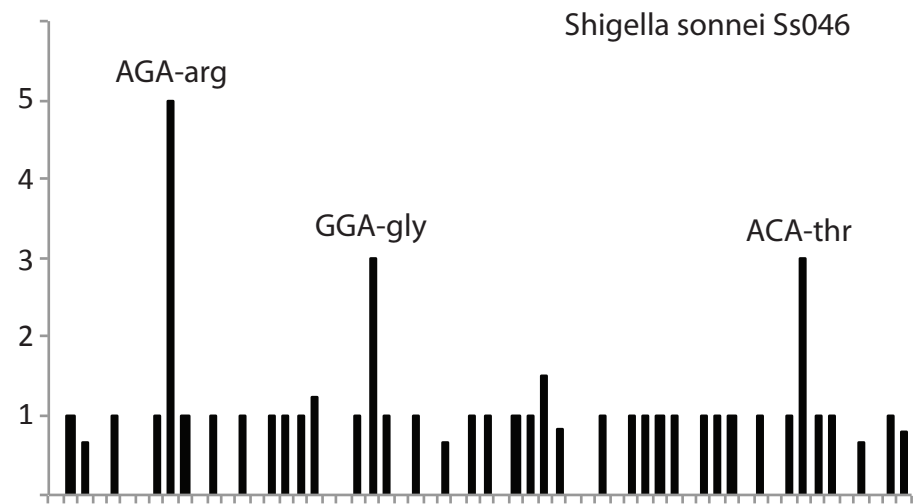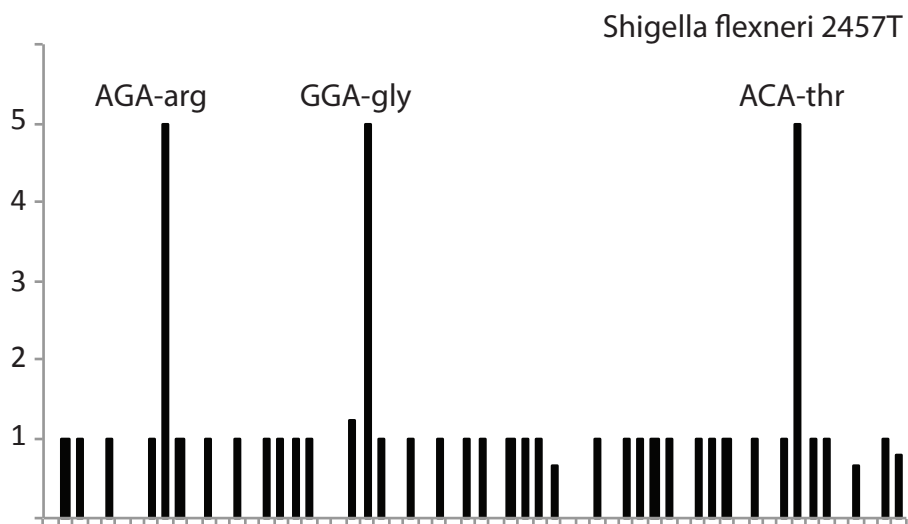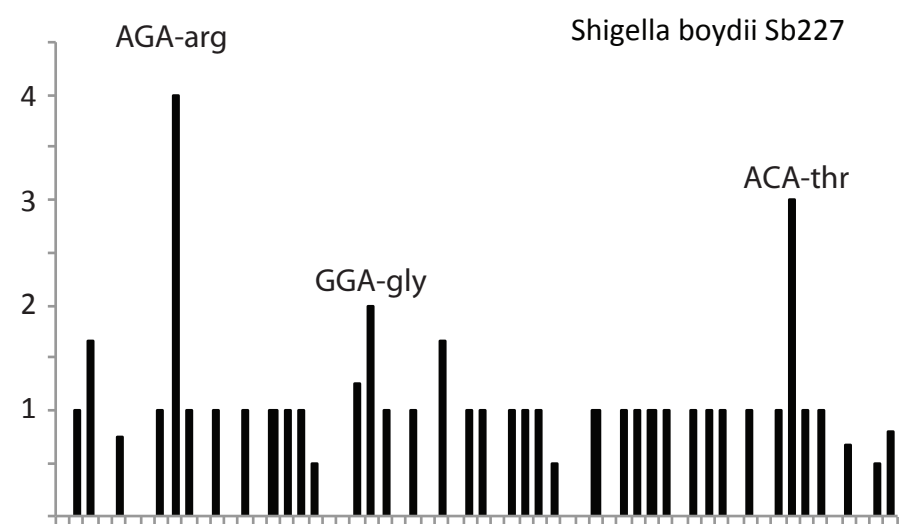

Supplement: Additional file 2: Figure S1. — Enriched tRNA genes for O157:H7 Edl933 concur with the tRNA genes enriched in O157:H7 Sakai (Fig. 2a). Enriched tRNA genes for all other Shigella strains concur with the tRNA genes enriched in O157:H7 Sakai (Fig. 2a). (PDF 847 kb) [file 12862_2015_441_MOESM2_ESM.pdf]
